# 1428. Increased Consumption of Pivmecillinam in Primary Care for Uncomplicated Urinary Tract Infection (uUTI) Is Not Associated With Increased Resistance Rates

**DOI:** 10.1093/ofid/ofab466.1620

**Published:** 2021-12-04

**Authors:** Anders Rhod Larsen, Anne Santerre Henriksen, Niels Frimodt-Møller

**Affiliations:** 1 Statens Serum Institut, Copenhagen, Hovedstaden, Denmark; 2 Maxel Consulting ApS, London, England, United Kingdom; 3 Rigshospitalet, Copenhagen, Hovedstaden, Denmark

## Abstract

**Background:**

The evolution of antibiotic resistance in Escherichia coli (E. coli) hampers the treatment of UTIs, mirroring the global public health concerns around antimicrobial resistance. Pivmecillinam, an oral prodrug of mecillinam (a β-lactam antibiotic), is used as first-line treatment for uUTIs in Denmark. Here, we examine the use of, and the prevalence of resistance to, mecillinam in Denmark in the primary care setting.

**Methods:**

Nationwide data on the use of and resistance to pivmecillinam (reported as its active form, mecillinam) was extracted and examined from the Danish Integrated Antimicrobial Resistance Monitoring and Research Programme (DANMAP) 2019 report (www.danmap.org). Prevalence estimates of resistance reported by DANMAP 2019 were obtained from the Danish Microbiology Database (MiBA).

**Results:**

In 2019, pivmecillinam accounted for about 27% of penicillins and 75% of penicillins with extended spectrum consumed in primary healthcare in Denmark. Pivmecillinam usage has increased primarily due to changes in recommendations for the treatment of uUTIs. Between 2010 and 2019, pivmecillinam usage in Denmark increased by 45% from 1.67 to 2.43, defined as daily doses per 1,000 inhabitants per day. In 2019, analysis of 83,850 urinary isolates from patients in the primary care setting with E. coli revealed a 5.3% resistance rate to mecillinam. Time-trend analysis using data from a 10-year period showed a small but significant decrease from the 5.5% resistance rate recorded in 2010 (p=0.001). In general, in spite of increasing use in Denmark, the development of resistance to pivmecillinam has remained low. In fact, a slight decline in pivmecillinam resistance was observed over the past decade.

**Conclusion:**

Despite the rising number of UTIs and the increasing use of pivmecillinam for uUTI in Denmark, over the past decade, the development of resistance to pivmecillinam remains low.

**Disclosures:**

**Anne Santerre Henriksen, MS**, **Advanz** (Consultant)**Shionogi BV** (Consultant)**UTILITY Therapeutics** (Consultant)

